# Arf1 GTPase Regulates Golgi‐Dependent G2/M Transition and Spindle Organization in Oocyte Meiosis

**DOI:** 10.1002/advs.202303009

**Published:** 2023-11-28

**Authors:** Kun‐Huan Zhang, Yuan‐Jing Zou, Meng‐Meng Shan, Zhen‐Nan Pan, Jia‐Qian Ju, Jing‐Cai Liu, Yi‐Ming Ji, Shao‐Chen Sun

**Affiliations:** ^1^ College of Animal Science and Technology Nanjing Agricultural University Nanjing 210095 China

**Keywords:** Arf1, Golgi, meiotic resumption, oocyte, spindle

## Abstract

ADP‐ribosylation factor 1 (Arf1) is a small GTPase belonging to the Arf family. As a molecular switch, Arf1 is found to regulate retrograde and intra‐Golgi transport, plasma membrane signaling, and organelle function during mitosis. This study aimed to explore the noncanonical roles of Arf1 in cell cycle regulation and cytoskeleton dynamics in meiosis with a mouse oocyte model. Arf1 accumulated in microtubules during oocyte meiosis, and the depletion of Arf1 led to the failure of polar body extrusion. Unlike mitosis, it finds that Arf1 affected Myt1 activity for cyclin B1/CDK1‐based G2/M transition, which disturbed oocyte meiotic resumption. Besides, Arf1 modulated GM130 for the dynamic changes in the Golgi apparatus and Rab35‐based vesicle transport during meiosis. Moreover, Arf1 is associated with Ran GTPase for TPX2 expression, further regulating the Aurora A–polo‐like kinase 1 pathway for meiotic spindle assembly and microtubule stability in oocytes. Further, exogenous Arf1 mRNA supplementation can significantly rescue these defects. In conclusion, results reported the noncanonical functions of Arf1 in G2/M transition and meiotic spindle organization in mouse oocytes.

## Graphical abstract

ADP‐ribosylation factor 1 (Arf1) GTPase plays a noncanonical role in mouse oocyte meiosis. Arf1 regulates G2/M transition during meiosis resumption, and this is based on its roles on Myt1 for Cyclin B1/CDK1 activity, and it also regulates tubulin acetylation and meiotic spindle organization which is due to its recruitment and transport of Aurora A‐Plk1.

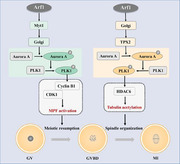

## 1 Introduction

Oocyte maturation is a unique asymmetric division composed of two rounds of meiosis, a prerequisite for successful fertilization and early embryo development in mammals.^[^ ^1^ ^]^ During the prophase of the first meiosis, fully grown oocytes are arrested in the germinal vesicle (GV) stage. Meiotic resumption marks the initiation of oocyte maturation, which is characterized by germinal vesicle breakdown (GVBD), followed by meiotic spindle assembly and migration during metaphase I (MI).^[^ ^2^ ^]^ Subsequently, cytokinesis occurs and the oocyte extrudes the first polar body (PBI) and is arrested in the metaphase II (MII) stage. The G2/M transition and microtubule dynamics are two key stages of oocyte maturation that facilitate asymmetric division.

Unlike mitosis, oocytes initiate the meiotic resumption (G2/M transition) from the first meiotic prophase arrest in response to a preovulatory surge of luteinizing hormone from the pituitary gland during each reproductive cycle in mammals, resulting in nonclassical patterns of GVBD.^[^ ^3^ ^]^ The G2/M transition is controlled by the activation of the maturation‐promoting factor (MPF).^[^ ^4^ ^]^ As a complex of core cell cycle regulators, MPF comprises catalytic subunit cyclin‐dependent kinase 1 (CDK1, also known as cyclin‐dependent kinase 2 (CDC2), and regulatory subunit cyclin B1.^[^ ^2^, ^5^ ^]^ Cyclin B1 regulates kinase activity by being synthesized and degraded at a specific time point during the cell cycle, while the transfer of cyclin B1 from the cytoplasm to the nucleus is the prerequisite for triggering GVBD.^[^ ^6^, ^7^ ^]^ The activation of CDK1 starts with binding to cyclin B1 and results from the balance between protein kinase Wee1/Myt1 and phosphatase CDC25.^[^ ^8^ ^]^ The phosphorylation of CDK1 at threonine 14 and tyrosine 15 catalyzed by Wee1/Myt1 leads to the inactivation of CDK1–cyclin B1 complex, whereas CDC25 inhibits phosphorylation in turn, thus promoting the occurrence of GVBD.^[^ ^9^ ^]^ After GVBD, the meiotic spindle assembly and chromosome congression lead to the MI stage. In mitotic cells, microtubules are primarily assembled from the centrosome, whereas in certain cells, such as mouse oocytes, typical centrosomes are absent. In these cells, centrosomes are eliminated and replaced by microtubule‐organizing centers for microtubule nucleation and function.^[^ ^10^, ^11^ ^]^ The absence of typical centrosomes in mouse oocytes leads to atypical patterns of spindle assembly. Reversible acetylation of tubulin is a post‐translational modification that affects spindle stability and formation by altering the microtubule structure.^[^ ^12^, ^13^ ^]^ Histone deacetylase 6 (HDAC6) is implicated in this reversible tubulin acetylation.^[^ ^14^ ^]^ Furthermore, the active form of Ran (Ran‐GTP) is required to induce microtubule nucleation and bipolar spindle assembly though reversing the inhibitory effect of importin‐α by spindle assembly factor TPX2.^[^ ^15^ ^]^ Bipolar spindle assembly and function are also under the control of protein phosphorylation that involves several protein kinases, such as serine/threonine (Thr) kinase Aurora A and polo‐like kinase 1 (Plk1).^[^ ^16^, ^17^ ^]^ The activity of Aurora A is regulated by autocatalytic phosphorylation at Thr288 and depends on cofactors such as Bora and TPX2, whereas the activation of Plk1 requires the phosphorylation at T210 by Aurora A.^[^ ^18^, ^19^ ^]^

ADP‐ribosylation factor (Arf) family comprises small GTPases containing Arf1‐6. Arf proteins function in the cycle of GDP‐bound inactive form and GTP‐bound active form, hydrolyzing GTP through GTP‐activating proteins and binding GTP through guanine nucleotide exchange factors.^[^ ^20^ ^]^ Emerging data indicate that Arf proteins participate in multiple biological processes, including membrane traffic, cytokinesis, endocytic recycle, lipid metabolism, and plasma membrane signaling.^[^ ^21^, ^22^, ^23^, ^24^ ^]^ Moreover, Arf proteins regulate actin and microtubule cytoskeleton dynamics by associating motor proteins to enable vesicle transport.^[^ ^25^, ^26^ ^]^ A previous study showed that the chlamydial effector InaC interacted with Arf1 to stabilize microtubules.^[^ ^27^ ^]^ For meiosis, Arf6 has been reported to participate in actin filament–mediated spindle movement and spindle formation via Arp2/3–cofilin pathway in mouse oocytes.^[^ ^28^ ^]^ Besides, both Arl2 (Arf‐like 2) and Arf5 maintain regular mitochondrial dynamicity, reactive oxygen species levels, and autophagy levels. They are also found to promote oocyte meiotic progression by affecting the cytoskeletal organization of microtubules and microfilaments.^[^ ^29^ ^]^ Although Arf1 localizes to the Golgi apparatus, endoplasmic reticulum–Golgi intermediate compartment and endosomes play a major role in the formation, budding, and fusion of COPI vesicles in retrograde and intra‐Golgi transport.^[^ ^30^, ^31^ ^]^ Recent research suggests that Arf1 recycles intra‐Golgi resident cargo and recruits necessary proteins to release secretory vesicles in the final stage of Golgi maturation.^[^ ^32^ ^]^ The activation of Arf1 prevents the recruitment of effectors to membranes. It blocks normal Golgi disassembly, leading to defects in coordinating various mitotic events, including incomplete chromosome segregation and cytokinesis furrow ingression.^[^ ^30^, ^33^ ^]^ Additionally, it was reported that expressing the dominant‐negative mutant form of Arf1 (Arf1^T31N^) induced actin filament assembly defects and caused symmetric cell division of oocytes.^[^ ^34^ ^]^

Although several studies have reported the multiple roles of Arf1 in different cellular processes, the mechanism by which Arf1 regulates mouse oocyte meiosis remains largely unclear. The present study was conducted to investigate the roles of Arf1 during mouse oocyte meiotic maturation using Arf1 depletion and rescue approaches. This study uncovered previously undescribed roles of Arf1 in meiotic resumption and spindle organization during mouse oocyte maturation, besides its function in Golgi‐mediated vesicle transport.

## 2 Results

### 2.1 Arf1 Modulated Meiotic Resumption and Polar Body Extrusion in Mouse Oocytes

**Figure 1:**
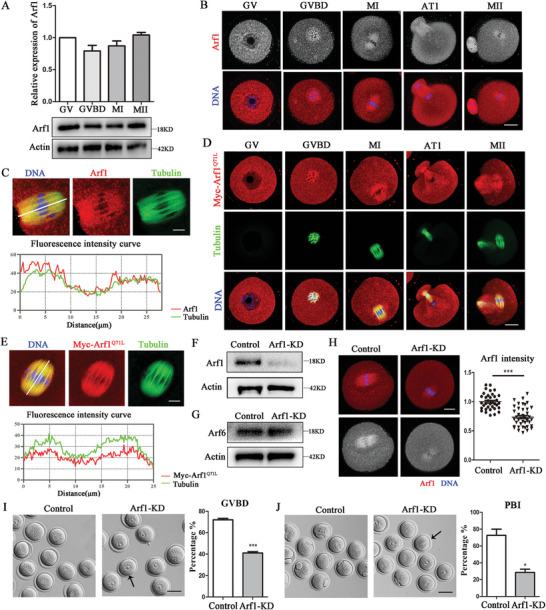
Arf1 modulated meiotic resumption and polar body extrusion in mouse oocytes. A) Arf1 protein levels in oocytes in different meiosis stages of GV (0 h), GVBD (4 h), MI (9 h), and MII (12 h) were examined by Western blot. Overall, 180 oocytes were used in each group. The quantitative analysis of the Arf1 protein band intensity indicated that Arf1 was expressed in all stages of meiotic maturation of mouse oocytes. B) Mouse oocytes from GV to MII stages were stained with anti‐Arf1 antibody and counterstained with Hoechst 33 342 to visualize DNA. Arf1 was localized in the cytoplasm, accumulated around the chromosomes in the GV stage, and enriched at microtubules from GVBD to MII stages. Red, Arf1; blue, DNA. Scale bar = 20 µm. C) Co‐staining of Arf1 and α‐tubulin in oocytes cultured to the MI stage. The graph shows the fluorescence intensity distributions of Arf1 and α‐tubulin along the white line. Red, Arf1; green, α‐tubulin; blue, DNA. Scale bar = 5 µm. D) Subcellular localization of Myc‐Arf1^Q71L^ during oocyte meiosis. The Myc‐Arf1^Q71L^ was detected in the cytoplasm during oocyte maturation, and Myc‐Arf1^Q71L^ was also accumulated around the chromosomes in the GV stage and localized with microtubules from GVBD to MII stage. Red, Myc‐Arf1^Q71L^; green, α‐tubulin; blue, DNA. Scale bar = 20 µm. E) Co‐staining of Myc‐Arf1^Q71L^ and α‐tubulin in oocytes cultured to the MI stage. The graph shows the fluorescence intensity distributions of Myc‐Arf1^Q71L^ and α‐tubulin along the white line. Red, Myc‐Arf1^Q71L^; green, α‐tubulin; blue, DNA. Scale bar = 5 µm. F) Western blot analysis showed a significant decrease in Arf1 protein level in the siRNA‐injection oocytes compared with the control oocytes. Overall, 180 oocytes were used in each group. G) Western blot analysis showed no difference in Arf6 expression between the control and Arf1‐KD groups. Overall, 180 oocytes were used in each group. H) Representative images of Arf1 distribution in the MI stage in the control and Arf1‐KD oocytes. The fluorescence intensity of the Arf1 signal significantly decreased after Arf1 depletion. Red, Arf1; blue, DNA. Scale bar = 20 µm. I) Representative images of GVBD in the control and Arf1‐KD oocytes. The oocytes with failed GV breakdown (black arrow). Scale bar = 80 µm. The rate of GVBD significantly decreased after Arf1 depletion. J) Representative images of polar body extrusion in the control and Arf1‐KD oocytes. The oocytes with no polar bodies. Scale bar = 80 µm (black arrow). Quantitative analysis showed that Arf1 depletion markedly reduced the rate of PBI. The data are presented as mean ± SEM from at least three independent experiments. ^*^ *p* < 0.05, ^***^ *p* < 0.001.

We First detected the expression and localization of Arf1 in different maturation stages of oocyte meiosis to investigate the roles of Arf1 in mouse oocytes. We collected Oocytes after culture for 0, 4, 9, and 12 h, corresponding to stages of GV, GVBD, MI, and MII, respectively. Arf1 was stably expressed in all stages of mouse oocyte meiosis (**Figure** **1**A). We Then detected the subcellular localization of Arf1 during mouse oocyte maturation. Our results indicated that Arf1 localized in the cytoplasm, accumulated around the chromosomes in the GV stage, and enriched in the microtubules from GVBD to MII stages (Figure 1B). The fluorescence intensity distribution confirmed that Arf1 tended to localize to the spindle (Figure 1C). Next, we investigated the localization patterns of GTP‐bound Arf1 during oocyte meiosis. The distribution of the active form of Myc‐Arf1^Q71L^ was found to be similar to that of Arf1 via mRNA microinjection (Figure 1D). The fluorescence intensity distribution also substantiated that Myc‐Arf1^Q71L^ tended to accumulate at the spindle (Figure 1E). We next examined the functions of Arf1 in mouse oocytes using the KD approach. After microinjection, Arf1 protein level significantly decreased in the Arf1‐KD group compared with the control group, which was confirmed by the band intensity analysis (control: 1.00 vs Arf1‐KD: 0.33 ± 0.02, *p* < 0.001) (Figure 1F). Additionally, Western blot results indicated no effect on Arf6 protein level after Arf1 depletion (Figure 1G). The fluorescence intensity analysis indicated that Arf1 signals in the Arf1‐KD oocytes were much lower than those in the control group (control: 1.00 ± 0.02, *n* = 35 vs Arf1‐KD: 0.74 ± 0.03, *n* = 41, *p* < 0.001; Figure 1H). A significant negative effect on meiotic resumption was observed in the Arf1‐depleted oocytes after a 3‐h culture (Figure 1I). The percentage of GVBD significantly reduced in Arf1‐depletion oocytes compared with control oocytes (control: 72.03 ± 1.28%, *n* = 251 vs Arf1‐KD: 41.00 ± 1.32%, *n* = 272, *p* < 0.001). Besides, we found that Arf1 depletion also disturbed the first polar body extrusion in mouse oocytes. The statistical analysis showed that the percentage of PBI was much lower in the Arf1‐KD group than in the control group (control: 72.60 ± 7.36%, *n* = 221 vs Arf1‐KD: 28.60 ± 3.75%, *n* = 211, *p* < 0.05; Figure 1J). These results indicated that Arf1 was essential for mouse oocyte meiotic maturation, and its depletion led to defects of meiotic resumption and polar body extrusion.

### 2.2 Arf1 Affected Myt1‐Mediated Cyclin B1/CDK1 Expression for Meiotic Resumption

**Figure 2:**
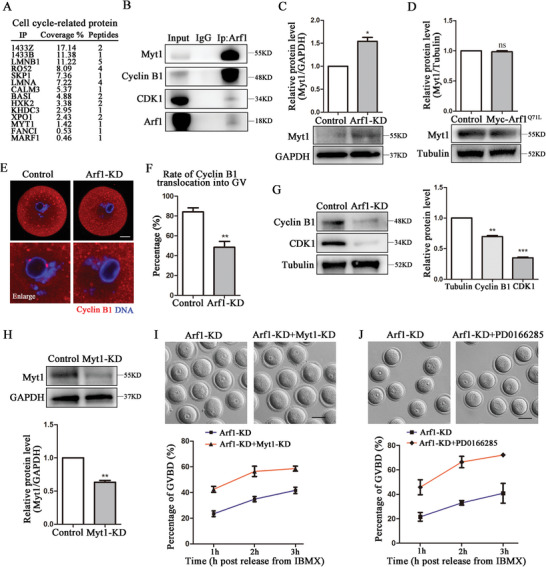
Arf1 affected Myt1‐mediated cyclin B1/CDK1 expression for meiotic resumption. A) Screening cell cycle‐related proteins connected with Arf1 by mass spectrometry analysis. B) Co‐IP results indicated that Arf1 was associated with protein kinase Myt1, cyclin B1, and CDK1. C) Expression of Myt1 in the control and Arf1‐depletion oocytes was determined by Western blot. Overall, 180 oocytes were used in each group. The quantitative analysis showed that the Myt1 protein level increased in the Arf1‐KD group. D) Western blot analysis showed no difference in Myt1 expression between the control and Myc‐Arf1^Q71L^ groups. Overall, 180 oocytes were used in each group. E) Representative images of cyclin B1 distribution in the GV stage in the control and Arf1‐KD oocytes. F) The rate of cyclin B1 translocation to GV decreased after Arf1 depletion. Red, cyclin B1; blue, DNA. Scale bar = 20 µm. G) Expression of cyclin B1 and CDK1 in the control and Arf1‐depletion oocytes was determined by Western blot. Overall, 180 oocytes were used in each group. The quantitative analysis showed that both cyclin B1 and CDK1 protein levels decreased in the Arf1‐KD group. H) Western blot analysis showed a significant decrease in the Myt1 protein level in the siRNA‐injection oocytes compared with the control oocytes. Overall, 150 oocytes were used in each group. I) Representative images of GVBD in the Arf1‐KD and Arf1‐KD+Myt1‐KD oocytes. The GVBD defect caused by Arf1 depletion was rescued after Myt1 depletion. Scale bar = 80 µm. J) Representative images of GVBD in the Arf1‐KD and Arf1‐KD+PD0166285 oocytes. The GVBD defect caused by Arf1 depletion was rescued after Wee1 inhibition. Scale bar = 80 µm. The data are presented as mean ± SEM from at least three independent experiments. ^*^ *p* < 0.05, ^**^ *p* < 0.01, ^***^ *p* < 0.001.

As the percentage of GVBD significantly decreased after deleting Arf1, we performed mass spectrometry analysis to explore the potential regulatory mechanism of Arf1 on oocyte meiotic resumption. We found that several cell cycle‐related proteins were associated with Arf1 including Myt1 (**Figure** **2**A). The immunoprecipitation results showed that Arf1 was bound with protein kinase Myt1, cyclin B1, and CDK1 (Figure 2B). Further analysis of the Western blot band intensity showed that Myt1 expression increased in the Arf1‐KD group at 40 min following the release from IBMX (control: 1.00 vs Arf1‐KD, 1.54 ± 0.09, *p* < 0.05; Figure 2C). However, Western blot results showed that the Myt1 protein level was not significantly changed after the injection of Myc‐Arf1^Q71L^ mRNA (Figure 2D), indicating that GTP‐bound Arf1 did not affect Myt1 activity. Then, we assessed the localization of cyclin B1. As a core cell cycle regulator, cyclin B1 localized to the nucleus in control GV oocytes; however, cyclin B1 signal weakened or dispersed from the nucleolus in Arf1‐depletion oocytes (Figure 2E). The rate of abnormal localization of cyclin B1 after Arf1 depletion significantly increased compared with that in the control group (control: 84.13 ± 4.05%, *n* = 37 vs Arf1‐KD: 48.43 ± 6.05%, *n* = 35, *p* < 0.01; Figure 2F). Moreover, both cyclin B1 and CDK1 protein levels decreased compared with those in the control group (cyclin B1: control, 1.00 vs Arf1‐KD, 0.70 ± 0.02, *p* < 0.01; CDK1: control, 1.00 vs Arf1‐KD, 0.35 ± 0.01, *p* < 0.001; Figure 2G). We examined the effects of Myt1 depletion on meiosis resumption to further confirm the effects of Arf1 on Myt1. We observed that Myt1 protein level significantly decreased in the Myt1‐KD group compared with the control group after microinjection (control: 1.00 vs Arf1‐KD, 0.63 ± 0.02, *p* < 0.01), which was confirmed by the band intensity analysis (Figure 2H). Myt1 depletion could recuse GV arrest caused by Arf1 depletion, which was reflected by the GVBD rate from statistical data after 3‐h culture (Figure 2I). Besides, we examined the effects of Wee1 kinase inhibitor PD0166285 on meiosis resumption. PD0166285 could also rescue GV arrest caused by Arf1 depletion (Figure 2J). These results indicated that Arf1 was crucial in MPF activation for mouse oocyte meiotic resumption.

### 2.3 Arf1 Participated in Golgi Apparatus Distribution During Mouse Oocyte Meiosis

**Figure 3:**
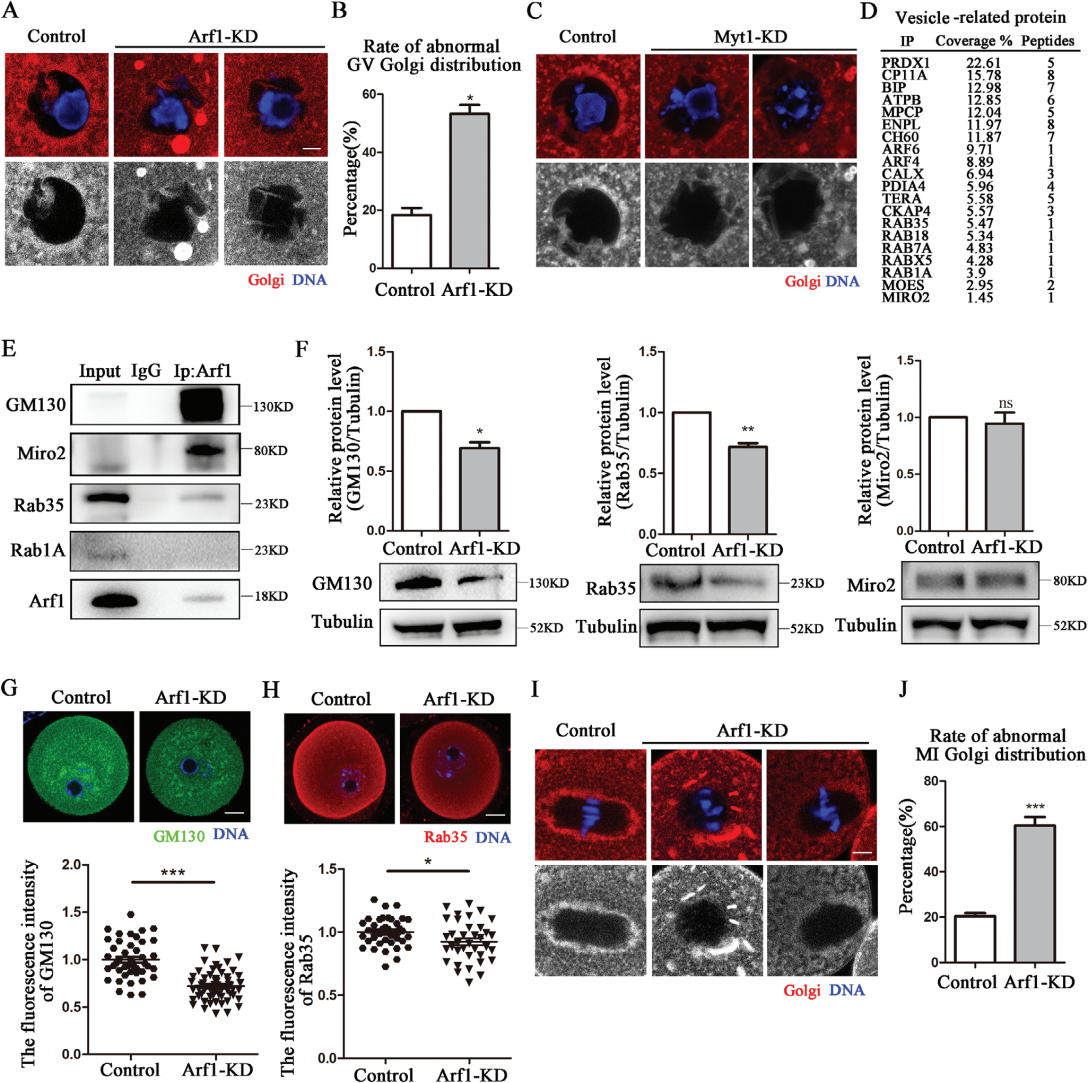
Arf1 participated in Golgi apparatus distribution during mouse oocyte meiosis. A) Representative images of Golgi distribution at the GV stage in the control and Arf1‐KD oocytes. Red, Golgi; blue, DNA. Scale bar = 5 µm. B) The rate of abnormal Golgi distribution in GV oocytes significantly increased after Arf1 depletion. C) Representative images of Golgi distribution in the GV stage in the control and Myt1‐KD oocytes. Red, Golgi; blue, DNA. Scale bar = 5 µm. D) Screening vesicle transport–related proteins connected with Arf1 by mass spectrometry analysis. E) Co‐IP results indicated that Arf1 was associated with *cis*‐Golgi marker GM130, GTPase Rab35, and Miro2, but not Rab1A. F) Western blot analysis showed a significant decrease in the GM130 and Rab35 protein levels in the Arf1‐KD oocytes compared with the control oocytes. Western blot analysis revealed no difference in Miro2 expression between the control and Arf1‐KD groups. Overall, 180 oocytes were used in each group. G) Representative images of GM130 distribution in the GV stage in the control and Arf1‐KD oocytes. The fluorescence intensity of the GM130 signal significantly decreased after Arf1 depletion. Green, GM130; blue, DNA. Scale bar = 20 µm. H) Representative images of Rab35 distribution in the GV stage in the control and Arf1‐KD oocytes. The fluorescence intensity of the Rab35 signal significantly decreased after Arf1 depletion. Red, Rab35; blue, DNA. Scale bar = 20 µm. I) Representative images of Golgi distribution in the MI stage in the control and Arf1‐KD oocytes. Red, Golgi; blue, DNA. Scale bar = 5 µm. J) The rate of abnormal Golgi distribution in MI oocytes significantly increased in the Arf1‐KD group compared with the control group. The data are presented as mean ± SEM from at least three independent experiments. ^*^ *p* < 0.05, ^**^ *p* < 0.01, ^***^ *p* < 0.001.

Golgi apparatus undergoes a multi‐step fragmentation process during cell division, whereas Arf1 is required for COPI vesicle formation and mitotic Golgi fragmentation.^[^ ^35^ ^]^ This prompted us to investigate the potential effect of Arf1 on the Golgi apparatus during mouse oocyte meiosis. Golgi apparatus was mainly localized around the GV in the GV stage of control oocytes; however, it was distributed in a clustered and agglutinated pattern in the Arf1‐KD group (**Figure** **3**A). The rate of abnormal Golgi distribution in GV oocytes significantly increased after Arf1 depletion (control: 18.40% ± 2.38%, *n* = 38 vs Arf1‐KD: 53.27% ± 3.11%, *n* = 49, *p* < 0.05; Figure 3B). Interestingly, the Golgi apparatus distribution after Myt1 depletion was similar to that in the Arf1‐KD group, suggesting a connection between Arf1, Myt1, and Golgi (Figure 3C). We analyzed mass spectrometry data and found several vesicle transport–related proteins, including Rab GTPase family, to explore the regulatory mechanism of Arf1 on Golgi apparatus distribution and vesicle transport (Figure 3D). We found through co‐IP experiments that Arf1 correlated with *cis*‐Golgi marker GM130, Rab35, and Miro2, but not with Rab1A (Figure 3E). Western blot results showed that the expression of GM130 and Rab35, but not Miro2, decreased after Arf1 depletion (GM130: control, 1.00 vs Arf1‐KD, 0.69 ± 0.05, *p* < 0.05; Rab35: control, 1.00 vs Arf1‐KD, 0.72 ± 0.03, *p* < 0.01) (Figure 3F). We subsequently stained the GV oocytes with *cis*‐Golgi marker GM130. The results showed that GM130 was distributed throughout the cytoplasm and concentrated around the GV in the control oocytes. Further, the fluorescence intensity analysis indicated that the intensity of GM130 signals in the Arf1‐KD oocytes was much lower than that in the control group (control: 1.00 ± 0.03, *n* = 44 vs Arf1‐KD: 0.72 ± 0.02, *n* = 60, *p* < 0.001; Figure 3G). The localization of Rab35 at the oocyte cortex and in the cytoplasm was also disturbed after Arf1 depletion, which was indicated by the intensity analysis (control: 1.00 ± 0.02, *n* = 37 vs Arf1‐KD: 0.92 ± 0.03, *n* = 36, *p* < 0.05; Figure 3H). Moreover, the Golgi apparatus in MI oocytes after Arf1 depletion was no longer evenly enriched in the spindle periphery area as observed in the control group, but dissipated or agglutinated in the cytoplasm (Figure 3I). The quantitative analysis demonstrated that the rate of abnormal Golgi distribution significantly increased in the Arf1‐KD group compared with the control group (control: 20.43% ± 1.43%, *n* = 39 vs Arf1‐KD: 60.53% ± 3.63%, *n* = 43, *p* < 0.001; Figure 3J). These results suggested that Arf1 associated with Myt1 to regulate Golgi apparatus distribution during mouse oocyte meiosis.

### 2.4 Arf1 Affected Tubulin Acetylation for Spindle Formation in Mouse Oocyte Meiosis

**Figure 4:**
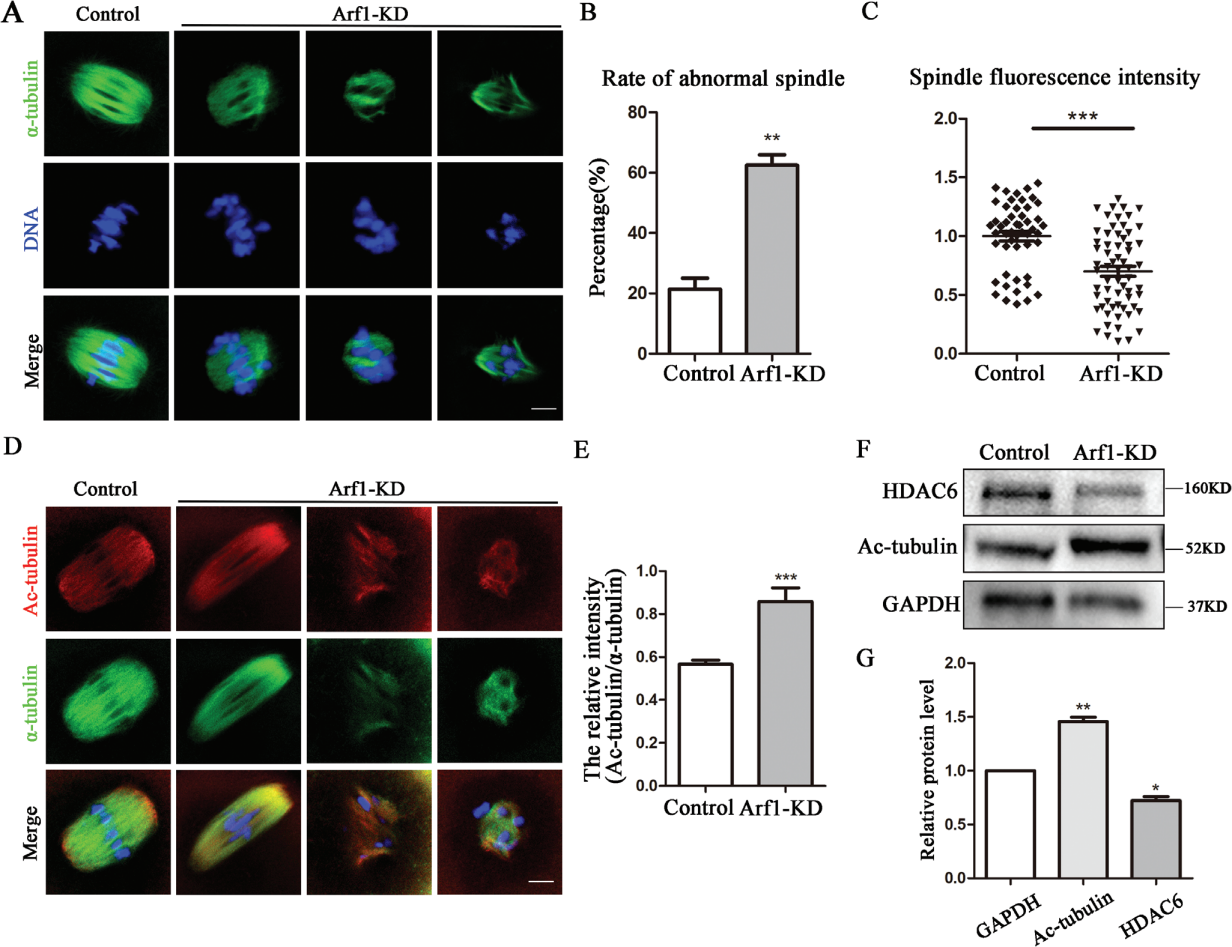
Arf1 affected tubulin acetylation for spindle formation in mouse oocyte meiosis. A) Representative images of spindle morphology at the MI stage in the control and Arf1‐KD oocytes. Green, α‐tubulin; blue, DNA. Scale bar = 5 µm. B) The rate of abnormal spindle distribution in MI oocytes significantly increased in the Arf1‐KD group compared with the control group. C) The fluorescence intensity of the spindle markedly decreased after Arf1 depletion. D) Representative images of acetylated α‐tubulin (ac‐tubulin) in the MI stage in the control and Arf1‐KD oocytes. Red, ac‐tubulin; Green, α‐tubulin; blue, DNA. Scale bar = 5 µm. E) The fluorescent signal ratio of ac‐tubulin to α‐tubulin increased in the Arf1‐KD group compared with the control oocytes. F) Expression of ac‐tubulin and deacetylase HDAC6 in control and Arf1‐depletion oocytes was determined by Western blot. Overall, 180 oocytes were used in each group. G) Quantitative analysis indicated that ac‐tubulin protein level significantly increased after Arf1 depletion, accompanied by decreased expression of HDAC6. The data are presented as mean ± SEM from at least three independent experiments. ^*^ *p* < 0.05, ^**^ *p* < 0.01, ^***^ *p* < 0.001.

Accurate spindle formation is essential for final polar body extrusion in oocytes. Given the localization of Arf1 on microtubules, we assessed the meiotic spindle morphology after Arf1 depletion. We observed that the oocytes in the control group had typical barrel‐shaped spindles with focused poles. In contrast, the spindles in the Arf1‐KD group had readily apparent defects, being smaller or fragmented. The microtubules were likely disassembled (**Figure** **4**A). The statistical analysis showed that the percentage of abnormal spindles after Arf1 depletion was significantly elevated compared with that in control oocytes (control: 21.43% ± 3.58%, *n* = 50 vs Arf1‐KD: 62.50% ± 3.48%, *n* = 74, *p* < 0.01; Figure 4B). Additionally, the immunofluorescence intensity of microtubules also significantly decreased (control: 1.00 ± 0.04, *n* = 50 vs Arf1‐KD: 0.70 ± 0.04, *n* = 63, *p* < 0.001; Figure 4C). We next determined the tubulin acetylation level to evaluate microtubule stability after Arf1 depletion. The immunostaining results showed that the fluorescent signal ratio of acetylated α‐tubulin (ac‐tubulin) to α‐tubulin in the Arf1‐KD group markedly increased compared with that in the control oocytes (Figure 4D); the fluorescence intensity analysis results substantiated our finding (control: 0.57 ± 0.02, *n* = 50 vs Arf1‐KD: 0.86 ± 0.06, *n* = 63, *p* < 0.001; Figure 4E). Moreover, the band intensity indicated that the ac‐tubulin protein level was much higher after Arf1 depletion, accompanied by decreased expression of deacetylase HDAC6 (Figure 4F), which was proved by statistical analysis (ac‐tubulin: control: 1.00 vs Arf1‐KD: 1.46 ± 0.04, *p* < 0.01; HDAC6: control: 1.00 vs Arf1‐KD: 0.72 ± 0.04, *p* < 0.05; Figure 4G). These data indicated that Arf1 regulated spindle formation and microtubule stability through its effects on tubulin acetylation during mouse oocyte maturation.

### 2.5 Arf1 Modulated Aurora A‐Plk1 for Spindle Assembly in Mouse Oocyte Meiosis

**Figure 5:**
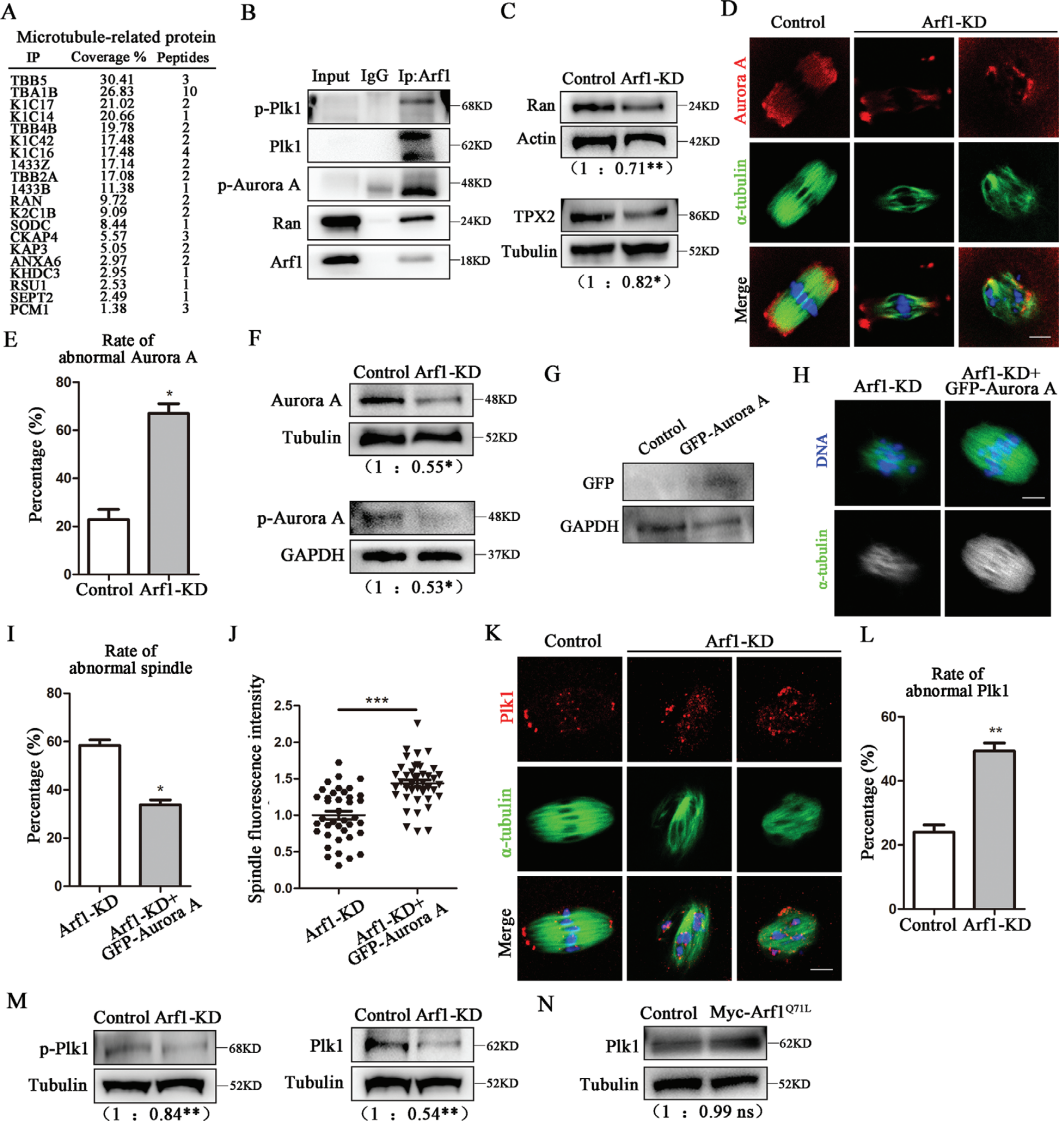
Arf1 modulated Aurora A–Plk1 for spindle assembly in mouse oocyte meiosis. A) Screening microtubule–related proteins connected with Arf1 by mass spectrometry analysis. B) Co‐IP results indicated that Arf1 was associated with Ran GTPase and protein kinases Aurora A and Plk1. C) Western blot analysis showed a significant decrease in Ran and TPX2 protein levels in the Arf1‐KD oocytes compared with the control oocytes. Further, 180 oocytes were used in each group. D) Representative images of Aurora A distribution in the MI stage in the control and Arf1‐KD oocytes. Red, Aurora A; Green, α‐tubulin; blue, DNA. Scale bar = 5 µm. E) The rate of abnormal Aurora A distribution significantly increased in the Arf1‐KD group compared with the control group. F) Western blot analysis showed that the expression of total Aurora A and p‐Aurora A significantly decreased after Arf1 depletion. Overall, 180 oocytes were used in each group. G) Protein expression of exogenous GFP–Aurora A was detected in the oocytes after mRNA injection. H) Representative images of spindle morphology in the MI stage in the Arf1‐KD and Arf1‐KD + GFP–Aurora A oocytes. Green, α‐tubulin; blue, DNA. Scale bar = 5 µm. I) Abnormal spindle rate caused by Arf1 depletion decreased by the supplementation of GFP–Aurora A mRNA. J) Quantitative analysis of spindle fluorescence intensity showed that GFP–Aurora A mRNA injection rescued the spindle defects after Arf1 depletion. K) Representative images of Plk1 distribution in the MI stage in the control and Arf1‐KD oocytes. Red, Plk1; green, α‐tubulin; blue, DNA. Scale bar = 5 µm. L) The rate of abnormal Plk1 distribution significantly increased in the Arf1‐KD group. M) Western blot analysis showed that both total Plk1 and p‐Plk1 protein levels significantly decreased after Arf1 depletion. Overall, 180 oocytes were used in each group. N) Western blot analysis showed no difference in Plk1 between the control and Myc‐Arf1^Q71L^ groups. Overall, 180 oocytes were used in each group. The data are presented as mean ± SEM from at least three independent experiments. ^*^ *p* < 0.05, ^**^ *p* < 0.01, ^***^ *p* < 0.001.

We performed mass spectrometry analysis to explore the regulatory mechanism of Arf1 on spindle assembly and found several microtubule‐related proteins including Ran GTPase (**Figure** **5**A). We found through co‐IP experiments that Ran GTPase and its downstream protein kinases Aurora A and Plk1 were bound with Arf1 (Figure 5B). We subsequently detected the expression level of Ran and its downstream target TPX2. In Arf1‐depleted oocytes, both Ran and TPX2 protein levels decreased compared with that in the control group (Ran: control: 1.00 vs Arf1‐KD: 0.71 ± 0.03, *p* < 0.01; TPX2: control: 1.00 vs Arf1‐KD: 0.82 ± 0.03, *p* < 0.05; Figure 5C). Then, we assessed the localization of Aurora A. As a well‐established regulator of spindle assembly, Aurora A localized to spindle poles in control MI oocytes; however, Aurora A signal weakened or dispersed from the abnormal spindles in Arf1‐depletion oocytes (Figure 5D). The rate of abnormal localization of Aurora A after Arf1 depletion significantly increased compared with that in the control group (control: 22.83% ± 4.22%, *n* = 36 vs Arf1‐KD: 66.93% ± 4.11%, *n* = 39, *p* < 0.05; Figure 5E). Moreover, the Arf1‐KD group displayed much lower protein level of Aurora A and its phosphorylation at Thr288 (p‐Aurora A) compared with that in the control group (Aurora A: control: 1.00 vs Arf1‐KD: 0.55 ± 0.06, *p* < 0.05; p‐Aurora A: control: 1.00 vs Arf1‐KD: 0.53 ± 0.05, *p* < 0.05; Figure 5F). Besides, we produced and injected exogenous GFP–Aurora A mRNA into the oocytes. GFP–Aurora A protein was expressed in oocytes after microinjection for 4 h (Figure 5G). We found that the abnormality of spindle morphology caused by Arf1 depletion could be partly rescued by GFP–Aurora A mRNA injection (Arf1‐KD: 58.43% ± 2.31%, *n* = 39 vs Arf1‐KD + GFP–Aurora A: 33.87% ± 1.96%, *n* = 44, *p* < 0.05; Figure 5H,I). Similarly, the microtubule fluorescence intensity caused by Arf1 depletion also could be partly rescued by GFP–Aurora A mRNA injection, which was confirmed by the statistical analysis data (Arf1‐KD: 1.00% ± 0.06%, *n* = 39 vs Arf1‐KD + GFP–Aurora A: 1.44% ± 0.05%, *n* = 41, *p* < 0.001; Figure 5J). Plk1 localized to spindle poles and centromeres in control MI oocytes, whereas Arf1 depletion resulted in an aberrant distribution of Plk1 displaying with the absence at spindle poles (Figure 5K). Further, the percentage of aberrant Plk1 localization after Arf1 depletion also markedly increased compared with that in the control oocytes (control: 24.03% ± 2.26%, *n* = 51 vs Arf1‐KD: 49.35% ± 2.48%, *n* = 63, *p* < 0.01; Figure 5L). Since Aurora A–mediated T210 phosphorylation of Plk1 (p‐Plk1) is critical for Plk1 kinase activity, we subsequently measured the expression level of total Plk1 and p‐Plk1. The analysis of the band intensity indicated that both Plk1 and p‐Plk1 protein levels significantly decreased after Arf1 depletion (Plk1: control: 1.00 vs Arf1‐KD: 0.54 ± 0.02, *p* < 0.01; p‐Plk1: control: 1.00 vs Arf1‐KD: 0.84 ± 0.01, *p* < 0.01; Figure 5M). Additionally, Western blot results revealed that the Myc‐Arf1^Q71L^ group displayed a similar Plk1 protein level compared with the control group (Figure 5N), indicating that GTP‐bound Arf1 did not affect Plk1. These results implied that Arf1 affected spindle assembly through the Aurora A–Plk1 pathway.

### 2.6 Exogenous Arf1 Supplement Rescued Oocyte Maturation Defects Caused by Arf1 Deletion

**Figure 6:**
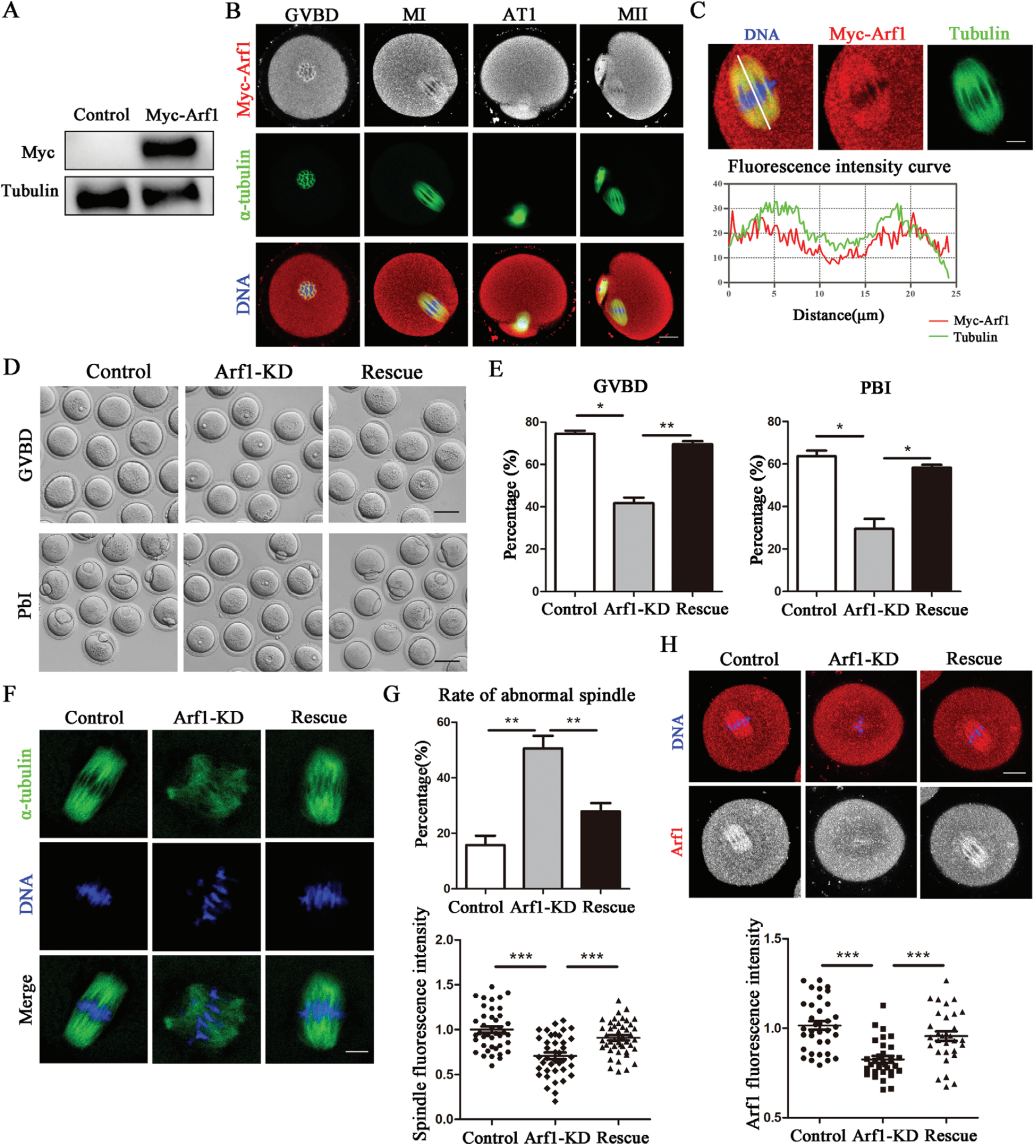
Exogenous Arf1 supplement rescued oocyte maturation defects caused by Arf1 deletion. A) Protein expression of exogenous Arf1 with a Myc tag was detected in the oocytes after the Myc‐Arf1 mRNA injection. B) Subcellular localization of Myc‐Arf1 during oocyte meiosis. Arf1 was accumulated around the chromosomes after GVBD and localized with microtubules in the oocytes from the MI to MII stages. Red, Myc‐Arf1; green, α‐tubulin; blue, DNA. Scale bar = 20 µm. C) Co‐staining of Myc‐Arf1 and α‐tubulin in oocytes cultured in the MI stage. The graph shows the fluorescence intensity distributions of Myc‐Arf1 and α‐tubulin along the white line. Red, Myc‐Arf1; green, α‐tubulin; blue, DNA. Scale bar = 5 µm. D) Representative images of GVBD and polar body extrusion in the control, Arf1‐KD, and rescue oocytes. Scale bar = 80 µm. The injection of Myc‐Arf1 mRNA rescued the GVBD and polar body extrusion defects caused by Arf1 depletion. Scale bar = 80 µm. E) Rate of GVBD and polar body extrusion in the control, Arf1‐KD, and rescue oocytes. F) Representative images of spindle morphology in the MI stage in the control, Arf1‐KD, and rescue oocytes. Green, α‐tubulin; blue, DNA. Scale bar = 5 µm. G) The abnormal spindle rate caused by Arf1 depletion decreased by the supplementation of Myc‐Arf1 mRNA. Quantitative analysis of spindle fluorescence intensity showed that Myc‐Arf1 mRNA injection rescued the spindle defects after Arf1 depletion. H) Representative images of Arf1 distribution in the MI stage in the control, Arf1‐KD, and rescue oocytes. Quantitative analysis of Arf1 fluorescence intensity indicated that Myc‐Arf1 mRNA injection rescued the Arf1 fluorescence intensity after Arf1 depletion. Red, Arf1; blue, DNA. Scale bar = 20 µm. The data are presented as mean ± SEM from at least three independent experiments. ^*^ *p* < 0.05, ^**^ *p* < 0.01, ^***^ *p* < 0.001.

We produced and injected exogenous Myc‐Arf1 mRNA into the oocytes to perform a rescue experiment. Myc‐Arf1 protein was expressed in oocytes after microinjection for 4 h (**Figure** **6**A). We collected oocytes in different meiotic maturation stages to reconfirm the subcellular localization of Arf1 in oocytes. Consistent with antibody staining results, Myc‐Arf1 localized in the cytoplasm, accumulated around the chromosomes in the GV stage, and enriched at microtubules from GVBD to MII stages (Figure 6B). The fluorescence intensity distribution also substantiated that Myc‐Arf1 tended to localize to the spindle (Figure 6C). Our results indicated that the injection of Myc‐Arf1 mRNA could rescue the defect of meiotic resumption and polar body extrusion in oocytes caused by Arf1 depletion (Figure 6D). Further, the rate of GVBD markedly increased compared with that in the Arf1‐KD group (control: 74.47% ± 1.45%, *n* = 180 vs Arf1‐KD: 41.70% ± 2.68%, *n* = 169, *p* < 0.05; Arf1‐KD vs rescue: 69.47% ± 1.49%, *n* = 157, *p* < 0.01). A similar finding was also reported for the rate of polar body extrusion (control: 63.63% ± 2.65%, *n* = 140 vs Arf1‐KD: 29.43% ± 4.74%, *n* = 157, *p* < 0.05; Arf1‐KD vs rescue: 58.23% ± 1.34%, *n* = 157, *p* < 0.05; Figure 6E). Additionally, we found that the abnormality of spindle morphology and microtubule fluorescence intensity could be partly rescued by Myc‐Arf1 mRNA injection (Figure 6F), which was confirmed by the statistical analysis of the data (abnormal spindle: control: 15.77 ± 3.37%, *n* = 37 vs Arf1‐KD: 50.60% ± 4.51%, *n* = 41, *p* < 0.01; Arf1‐KD vs rescue: 27.90% ± 2.96%, *n* = 39, *p* < 0.01) (fluorescence intensity: control: 1.00% ± 0.04%, *n* = 41 vs Arf1‐KD: 0.71% ± 0.04%, *n* = 39, *p* < 0.001; Arf1‐KD vs rescue: 0.91% ± 0.03%, *n* = 44, *p* < 0.001) (Figure 6G). The results of statistical analysis of the data in this study also revealed that the injection of Myc‐Arf1 mRNA could rescue the Arf1 fluorescence intensity (control: 1.02% ± 0.02%, *n* = 35 vs Arf1‐KD: 0.83% ± 0.02%, *n* = 31, *p* < 0.001; Arf1‐KD vs rescue: 0.96% ± 0.03%, *n* = 31, *p* < 0.001; Figure 6H).

## 3 Discussion

**Figure 7:**
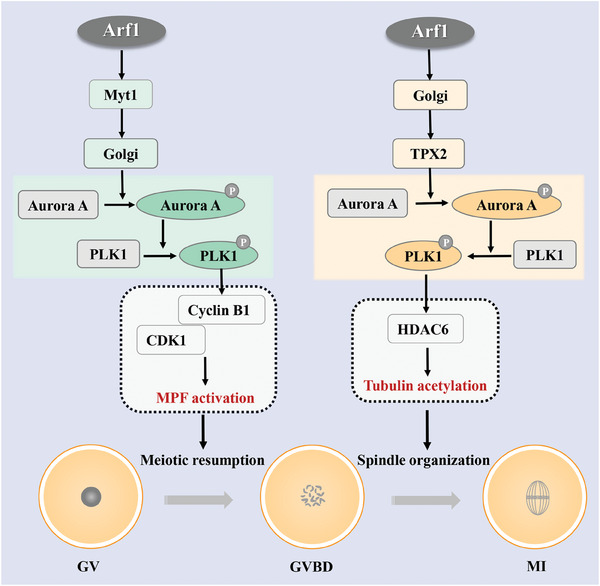
Roles of Arf1 during mouse oocyte meiosis. Arf1 affected Myt1 activity for cyclin B1/CDK1‐based G2/M transition. Besides, Arf1 modulated GM130 for the dynamic changes in the Golgi apparatus and Rab35‐based vesicle transport during meiosis. Moreover, Arf1 was associated with Ran GTPase for TPX2 expression, which further regulated the Aurora A–Plk1 pathway for meiotic spindle assembly and microtubule stability in oocytes.

A small GTPase Arf1 has been reported to function in mitotic vesicle transport and other cellular events. This study focused on the potential regulatory roles and mechanisms of Arf1 in mouse oocyte meiosis. We found that compared with the somatic cells, Arf1 played novel functions in meiotic resumption, microtubule stability, and spindle organization during oocyte maturation, which revealed previously undescribed critical roles of Arf1 in mouse oocyte meiosis (**Figure** **7**).

The first major finding was that Arf1 was involved in G2/M transition during mouse oocyte meiosis, showing the failure of GVBD and the aberrant expression of cyclin B1/CDK1 and Myt1. MPF is a cell cycle–regulating complex for G2/M transition in oocyte meiosis, which is formed by cyclin B1 and CDK1,^[^ ^36^ ^]^ whereas protein kinase Myt1 negatively regulates CDK1–cyclin B1 complexes and the overproduction of Myt1 can interfere with cell cycle progression.^[^ ^37^ ^]^ MPF enters the nucleus and phosphorylates nuclear pore complexes to ensure nuclear envelope breakdown, whereas Arf1 mediates the binding of nuclear‐specific vesicles to chromatin and subsequent vesicle fusion–mediated double nuclear membrane surrounding chromatin, which further affects nuclear envelope assembly during mitosis.^[^ ^38^ ^]^ These facts suggest that Arf1 may regulate Myt1 to ensure nuclear membrane disassembly for germinal vesicle breakdown in oocytes. Moreover, Myt1 controls intracellular membrane dynamics during mitosis,^[^ ^39^ ^]^ as evidenced by its regulation of Golgi fragmentation in the G2 phase and Golgi breakdown into tubules and vesicles in mitosis.^[^ ^40^ ^]^ The block in Golgi fragmentation inhibits Aurora A recruitment and activation at the centrosome. This, in turn, along with Plk1, impairs the activation of Cdk1–cyclin B1 in the early G2 phase and the onset of mitosis, ultimately resulting in the failure of progression to mitosis.^[^ ^41^ ^]^ Based on mitosis, we believed that Golgi also regulated MPF activity for G2/M transition. This led us to speculate that the failure of meiotic resumption upon Arf1 depletion in mouse oocytes was due to abnormal Golgi fragmentation mediated by Myt1. Further, our results indicated the block in Golgi fragmentation after Arf1 depletion, which proved our hypothesis. Arf1 drives the assembly of different coats onto budding vesicles, which are closely connected with the Golgi apparatus.^[^ ^42^ ^]^ Similarly, our data indicated that Arf1 was associated with several vesicle transport‐related proteins, and Arf1 affected the localization and expression of *cis*‐Golgi marker GM130 and GTPase Rab35. Indeed, the dominant‐negative Arf1 mutant in myotubes alters the subcellular distribution of GM130, which impairs endoplasmic reticulum–Golgi traffic.^[^ ^43^ ^]^ Arf1 is also a master regulator that coordinates Rab GTPase conversion at the trans‐Golgi network in yeast.^[^ ^44^ ^]^ Therefore, our data indicated that Arf1 modulated Myt1 for the Golgi apparatus, which further activated MPF for meiosis resumption in oocytes.

Moreover, we found the misdistribution of the Golgi apparatus in MI‐stage oocytes after Arf1 depletion. In contrast, the dynamic changes in the Golgi apparatus in terms of intracellular transport were closely related to microtubules.^[^ ^45^ ^]^ Besides, we found that Arf1 localized in the cytoplasm, accumulated around the chromosomes in the GV stage, and enriched at microtubules from GVBD to MII stages in the mouse oocyte meiosis. This localization pattern was not consistent with that of somatic cells during mitosis. For example, Arf1 localizes at the Golgi membrane in HeLa cells and normal rat kidney cells.^[^ ^33^, ^46^ ^]^ These facts prompted us to explore the effects of Arf1 on the meiotic spindle during oocyte meiosis. Our results showed that Arf1 depletion affected microtubule dynamics and meiotic spindle morphology, which was not reported in mitosis. A previous report in meiosis also indicated that Arf1 regulated spindle rotation and actin organization by regulating MAP kinase activity, which in turn affected cytokinesis.^[^ ^34^ ^]^ However, the abnormal pattern of spindle morphology was slightly different, and this difference might be caused by the use of different experimental methods between the dominant‐negative mutant form of Arf1 (Arf1^T31N^) and knockdown/rescue. Besides, Arf5 and Arf6 are involved in spindle formation during mouse oocyte meiosis, indicating that the Arf GTPase family has general but different roles in microtubule dynamics in oocyte meiosis compared with mitosis.^[^ ^28^, ^29^ ^]^ Microtubules have been implicated in various covalent modifications, including reversible tubulin acetylation.^[^ ^47^ ^]^ Tubulin acetylation confers resilience to mechanical stress on the microtubules, further regulating microtubule stability.^[^ ^48^ ^]^ Further, our data indicated that Arf1 regulated tubulin acetylation levels to modulate microtubule stability in mice, which could be confirmed by the altered expression of HDAC6 since deacetylase HDAC6‐mediated deacetylation regulated microtubule‐dependent cell motility.^[^ ^14^ ^]^

The activation of Aurora A and Plk1, keys to bipolar spindle formation, is essential for HDAC6 to induce its deacetylase activity in primary cilia.^[^ ^49^, ^50^ ^]^ Further, both Aurora A KD and Plk1 inhibition interfere with meiotic resumption and proper spindle assembly in mouse oocytes.^[^ ^51^, ^52^ ^]^ We next investigated the mechanism of Arf1 on spindle assembly. We found that Arf1 was associated with Aurora A and Plk1, and their phosphorylation levels were altered. The spindle assembly factor TPX2 is crucial for activating Aurora A kinase and stimulating microtubule nucleation. TPX2 is released from the inhibitory binding of importin‐α by the activated form of Ran.^[^ ^53^ ^]^ However, Arf1 depletion disturbed the expression of Ran and TPX2, indicating that Arf1 regulated Ran and TPX2 for meiotic spindle formation in oocytes. Interestingly, the Golgi matrix protein GM130 interacted with importin‐α, sequestered importin‐α from TPX2 to Golgi membranes during mitotic entry, and liberated TPX2 to activate Aurora A.^[^ ^54^ ^]^ Moreover, *cis*‐Golgi protein p28 had an influence on the fragmentation of the Golgi and the amount of acetylated tubulin, further indicating that Golgi organization could affect acetylation in microtubules.^[^ ^55^ ^]^ All these data suggested the critical roles of Arf1 GTPase on meiotic spindle assembly in mouse oocyte meiosis.

In conclusion, our results demonstrated the unreported roles of Arf1 on the Myt1‐based Golgi apparatus to regulate MPF activity for meiotic resumption and spindle organization via the Aurora A–Plk1 pathway during mouse oocyte meiosis.

## 4 Experimental Section

### Antibodies and Chemicals

Anti‐Arf1 antibody (#10790‐1‐AP and #20226‐1‐AP), anti‐Rab35 antibody (#11329‐2‐AP), anti‐HDAC6 antibody (#16167‐1‐AP), anti‐NAT10 antibody (#13365‐1‐AP), anti‐Ran antibody (#10469‐1‐AP), anti‐TPX2 antibody (#11741‐1‐AP), anti‐Myt1 antibody (#67806‐1‐lg), anti‐GAPDH antibody (#60004‐1‐lg), and anti‐α‐tubulin antibody (#11224‐1‐AP) were purchased from Proteintech (Rosemont, IL, USA). Anti‐GM130 antibody (#DF7556) was purchased from Affinity Biosciences (Cincinnati, OH, USA). Anti‐Aurora A antibody (#AF1708) was purchased from Beyotime (Shanghai, China). Anti‐Plk1 antibody (#37‐7000) was purchased from Invitrogen (Carlsbad, CA, USA). Anti‐Ac‐tubulin antibody (#T7451), anti‐α‐tubulin‐FITC antibody (#F2168), and Hoechst 33 342 were purchased from Sigma–Aldrich (St. Louis, MO, USA). Anti‐Myc tag (#ab18185), Alexa Fluor 594 Goat Anti‐Rabbit IgG (H + L) (#ab150080), Alexa Fluor 594 Goat Anti‐Mouse IgG (H + L) (#ab150116), anti‐CDK1 antibody (#ab18), anti‐cyclin B1 antibody (#ab181593), and anti‐GM130 antibody (#ab52649) were purchased from Abcam (Cambridge, UK). Anti‐phospho‐Aurora A (Thr288)/Aurora B (Thr232)/Aurora C (Thr198) antibody (#2914T) and antiactin antibody (#3700) were purchased from Cell Signaling Technology (Danvers, MA, USA). Horseradish peroxidase–conjugated goat antirabbit/mouse antibodies (CW0103/CW0102) were purchased from CWBIO (Beijing, China).

### Oocyte Collection and Culture In Vitro

All experimental procedures were conducted in accordance with the institutional guidelines of the Animal Research Ethics Committee of Nanjing Agricultural University, China. This study was approved by the experimental animal committee of Jiangsu Province (SYXK‐Su‐20170007). Female Institute of Cancer Research (ICR) mice (4–6 weeks old) were housed in a room with a controlled temperature of 22 °C and fed a regular diet. Fully grown GV‐stage oocytes were collected from the ovaries of ICR mice, cultured in M2 medium with paraffin oil at 37 °C and in the presence of 5% CO_2_ for in vitro maturation. In specific stages, the oocytes were collected for different detections and analyses.

### Microinjection of Arf1 siRNA and mRNA

Arf1 siRNA (Santa Cruz, sc‐141186) was dissolved in RNase‐free water to make a 50‐µm working solution. For Arf1 knockdown (KD), 5–10 pL siRNA was microinjected into the cytoplasm of GV‐stage oocytes, while an equal volume of negative control was microinjected for the control group. Then, the oocytes were arrested in the GV stage for at least 20 h in an M2 medium supplemented with 100 µm 3‐Isobutyl‐1‐methylxanthine (IBMX) to maximize the siRNA efficiency and facilitate the depletion of Arf1. Then, the oocytes were washed six times and cultured at different time points in a fresh M16 medium.

Further, 5–10 pL of 900 ng µL^−1^ Myc‐Arf1 mRNA was directly injected into the GV oocytes for verifying protein expression and 400 ng µL^−1^ Myc‐Arf1 mRNA for localization detection. For the rescue experiment, 5–10 pL of 400 ng µL^−1^ Myc‐Arf1 mRNA was injected into the GV oocytes 20 h after Arf1 siRNA injection. Next, the GV oocytes were cultured in an M16 medium supplemented with 100‐µm IBMX for 4 h. Then, the oocytes were washed six times and cultured in fresh M16 medium for subsequent experiments.

### Plasmid Construction and mRNA Synthesis

Myc‐Arf1 and Myc‐Arf1^Q71L^ were constructed by Wuhan GeneCreate Biological Engineering Co., Ltd. an HiScribe T7 High‐Yield RNA Synthesis Kit was used (New England Biolabs, Ipswich, UK) to synthesize mRNA from the plasmids linearized by SMAI, capped with m^7^G(5′)ppp(5′)G RNA Cap Structure Analog (New England Biolabs), and tailed with Poly(A) using a Polymerase Tailing Kit (Epicentre, Madison, WI, USA) and then purified using an RNA Clean & Concentrator Kit (Zymo Research, Irvine, CA, USA). Finally, the Arf1 mRNA and Arf1^Q71L^ mRNA were preserved at −80 °C.

### Immunofluorescence Staining and Confocal Microscopy

The oocytes were fixed with 4% paraformaldehyde for 30 min and permeabilized with 0.5% Triton X‐100 in phosphate‐buffered saline (PBS) for 20 min at room temperature. After blocking in PBS supplemented with 1% bovine serum albumin for 1 h, the oocytes were incubated with primary antibodies at 4 °C overnight. Next, the oocytes were washed three times (3 min each) in PBS containing 0.1% Tween 20 and 0.01% Triton X‐100 and then incubated with appropriate secondary antibodies for 1 h at room temperature. For the staining of spindles, the oocytes were incubated with a direct‐fluorescence anti‐α‐tubulin‐FITC antibody at 4 °C overnight. Hoechst 33 342 (10 µg mL^−1^) was used to stain chromosomes for 15 min at room temperature. Finally, a laser‐scanning confocal microscope was used (Zeiss LSM 800 META, Germany) to observe the oocytes.

Live‐cell fluorescence staining was used to detect the distribution of the Golgi apparatus. GV‐ and MI‐stage oocytes were cultured in an M16 medium supplemented with 1% protease for 3 min to degrade the zona pellucida of oocytes. After washing three times in the M2 medium, the oocytes were transferred to Golgi‐Tracker Red (C1043‐1; Beyotime) diluted with M16 medium at 4 °C for 30 min. Hoechst 33 342 diluted with M16 medium was used to stain chromosomes for 30 min at 37 °C and in the presence of 5% CO_2_, and then the oocytes were immediately imaged under the confocal microscope.

### Fluorescence Intensity Analysis

Immunofluorescence was performed in parallel and under identical conditions in the control and treatment groups. The images were always obtained using the same confocal microscope settings. The average fluorescence intensity per unit area of the target area was measured after fluorescence staining. The fluorescence intensity of the samples was analyzed using ZEN lite 2012 and ImageJ software (National Institutes of Health, MD, USA).

### Co‐Immunoprecipitation and Western Blot Analysis

The samples of 500 µL ovarian lysate incubated with Arf1 antibody and bead complexes were sent to BGI‐Shenzhen Co., Ltd. (China) for mass spectrometry (MS) analysis. Protein gel strips were identified by separating the sample proteins by gel electrophoresis. Then, the protein gel strips were obtained at different positions on the film and extracted the peptides after enzymatic digestion. Next, mass spectrometry was used to obtain the mass spectrum of the proteins in these gel strips. Finally, the protein identification software was used to identify the proteins in the samples. The protein identification used experimental MS/MS data and aligned them with theoretical MS/MS data from the database. The National Center for Biotechnology Information (NCBI) was used for retrieval and analysis of the data.

For co‐IP, 10 ovaries were harvested into a lysis buffer containing a protease inhibitor cocktail and thoroughly ground on ice. The rabbit polyclonal anti‐Arf1 and rabbit IgG antibodies were incubated with the cell lysate at 4 °C overnight and subsequently incubated with Dynabeads Protein G (Thermo Fisher Scientific, Waltham, MA, USA) at 4 °C for 5 h. Then, the tubes were placed on a magnet. After washing three times, the immune complexes were released from the beads by mixing 2 × SDS loading buffer at 30 °C for 10 min. NuPAGE LDS sample buffer (Thermo Fisher Scientific) was subsequently added to the samples, followed by heating at 100 °C for 10 min, and then stored at−20 °C.

Approximately 180 mouse oocytes were collected in NuPAGE LDS sample buffer at 100 °C for 10 min and rapidly frozen at −20 °C for Western blotting. The samples were treated with 10% sodium dodecyl sulfate–polyacrylamide gel electrophoresis at 160 V for 1 h and then transferred to polyvinylidene fluoride (PVDF) membranes at 20 V for 90 min. The PVDF membranes were then blocked in QuickBlock Blocking Buffer for 30 min at room temperature and incubated at 4 °C overnight with primary antibodies. After washing three times with Tris Buffered Saline with Tween 20 (TBST) for 10 min each, the membranes were incubated with horseradish peroxidase–conjugated Pierce Goat anti‐rabbit or mouse IgG (1:1000) for 1 h at room temperature. The specific proteins were finally visualized using a high‐quality ECL Western blotting system (Tanon, Shanghai, China), and the band intensity values were quantified using ImageJ software. A blot was stripped and reprobed several times to visualize other proteins or optimize protein detection. The Restore PLUS Western Blot Stripping Buffer (#46430; Thermo Fisher Scientific) was used to remove the primary and secondary antibodies from a Western blot so that the blot could be reprobed.

### Statistical Analysis

Each experiment had at least three biological replicates. The data were analyzed using GraphPad Prism five statistical software (GraphPad, CA, USA) by the *t*‐test to compare the statistical significance between the control and treatment groups. The data were expressed as mean ± standard error of the mean (SEM). A *p* value <0.05 indicated a statistically significant difference.

## Conflict of Interest

The authors declare no conflicts of interest.

## Data Availability

The data that support the findings of this study are available from the corresponding author upon reasonable request.
